# Experiences in the training of specialist family and community nurses: a qualitative study

**DOI:** 10.3389/fpubh.2023.1154084

**Published:** 2023-05-04

**Authors:** Francisca Sánchez-Muñoz, Isabel María Fernández-Medina, José Granero-Molina, Iván Claudio Suazo-Galdames, Susana Nunez-Nagy, María Isabel Ventura-Miranda, María Dolores Ruíz-Fernández

**Affiliations:** ^1^Health Care Centre San Vicente del Raspeig 1, Department of Alicante, Alicante, Spain; ^2^Department of Nursing, Physiotherapy and Medicine, University of Almería, Almería, Spain; ^3^Facultad de Ciencias de la Salud, Universidad Autónoma de Chile, Temuco, Chile; ^4^Department of Physiotherapy, University of Alcalá, Alcalá de Henares, Madrid, Spain

**Keywords:** primary care, family and community nurse, specialist nurse, training, experiences, qualitative study

## Abstract

**Introduction:**

The specialist Family and Community Nurse Practitioner (FCNP) is a professional who, after a period of training, is qualified to be part of multidisciplinary teams in primary care. The aim of this study was to describe and understand the experiences of nurses during their training process in the specialty of Family and Community Nursing in Spain.

**Methods:**

A descriptive qualitative study was carried out. Participants were recruited by means of convenience sampling from January to April 2022. Sixteen specialist nurses in Family and Community Nursing from different autonomous communities in Spain participated in the study. Twelve individual interviews and one focus group were conducted. Data were analyzed following a thematic analysis method in ATLAS.ti 9.

**Results:**

The results showed two themes and six subthemes: (1) Residency period, more than a training: (a) Training during the residency period; (b) Specializing through a constant struggle; (c) Moderate optimism about the future of the specialty; and (2) A journey from illusion to disappointment: (a) Beginning of the residency: feeling special; (b) During the residency: between satisfaction and misunderstanding; (c) At the end of the residency: power and frustration.

**Conclusions:**

The residency period is important in the training and acquisition of competencies for the Family and Community Nurse Practitioner. Improvements are needed to ensure quality training during residency and to help give visibility to the specialty.

## 1. Introduction

The World Health Organization (WHO) describes the Family and Community Nurse Practitioner (FCNP) as the professional who, through a comprehensive and holistic vision, takes on the mission of accompanying people from birth to death to develop their health potential, promoting the different family, work, and social environments to facilitate this development (1, 2). These professionals are prominent components of multidisciplinary primary care teams alongside other health professionals (3).

Internationally, several common competencies and/or standards have been developed to define the role and articulate the value of FCNPs in primary care practice (4–6). Although there is ambiguity in the title or appointment of this professional, the training process in the acquisition of competencies generally varies from 2 to 4 years depending on the regulations established in each country (7). In Spain, after the 4-year nursing degree, the specialist nurse is trained after passing an entrance exam to the specialty, in accordance with Article 20.2 of the Law on the Management of Health Professions (2003). There are several specialties regulated in Royal Decree 450/2005 of 22 April 2005: Occupational Nursing, Geriatrics, Pediatrics, Obstetrics and Gynecology (midwifery), Mental Health and FCNP (8). The training for the FCNP specialty is as Resident Internal Nurse (RIN) together with Family and Community doctors in Multiprofessional Teaching Units accredited by the Ministry of Education (8).

In the Order SAS/1729/2010, of 17 June, which approves and publishes the training programme for the specialty of Family and Community Nursing, resident nurses will be tutored by a tutor during the 2 years of training. At least 4 months each year must be spent in the same health center as their tutor and the rest of the time they may rotate through other specific services or devices decided by the teaching committee of the multidisciplinary units. In this training they are instructed to work within interdisciplinary primary care teams and other hospital and community facilities (9). Their competencies are linked to the provision of advanced care in clinical family and community care to individuals throughout their life cycle and families; public and community health; epidemiological surveillance; teaching; management of care and services in family and community settings; and research in the area of nursing (6, 10).

The WHO in one of its reports highlighted the importance of investing in the training of nurses, emphasizing the contribution made by the nursing profession and confirming that investing in it is a benefit to society rather than a cost (11). Similarly, there is evidence linking the specialist nurse to increased quality of care (12). The integration of FCNP specialists has been associated with increased quality of care at the family and community level, in addition to a reduction in healthcare costs, as they are able to deliver person-centerd care incorporating research and evidence-based practice (13, 14). They are thus considered a valuable resource in healthcare systems as they have the knowledge, competencies, and skills for health promotion and prevention, acute and chronic disease management, in addition to supporting the work of the entire primary care team (15, 16).

Despite the competencies assumed by FCNPs, studies that have analyzed the experiences of these nurses in the training they receive are practically scarce in Spain and internationally (17). Analyzing their experiences could help health system organizations to guide their training and take advantage of this valuable resource with a consequent improvement in the quality of care (5). For this reason, the aim of this study was to describe and understand the experiences of nurses during their training process in the specialty of Family and Community Nursing in Spain.

## 2. Materials and methods

### 2.1. Design

A descriptive qualitative study was conducted (18). This design allows us to describe the felt reality of the participants from a closer perspective. It is therefore possible for us to learn about the experiences regarding the training of the specialty of FCNPs from the perspective of specialist nurses in Spain. The quality standards of the COREQ guide (19) were followed (Supplementary File 1).

### 2.2. Participants

The study was carried out between January 2022 and April 2022. Participants were recruited by convenience sampling through social networks due to the ease and availability of access to participants. To this end, a total of 233 nurses specializing in FCNP from all over Spain were invited by means of a letter via email. The inclusion criteria were: (1) to be a specialist nurse in family and community nursing; and (2) to have completed the training period in the last 5 years. The exclusion criteria were: (1) failure to reply to the study invitation e-mail; (2) failure to sign the informed consent form; and (3) not speaking Spanish. 191 specialist nurses did not respond to the invitation. 42 participants replied to the email, of whom 26 did not give their consent to participate in the study. In the end, a total of 16 FCNPs from different autonomous communities in Spain with a mean age of 29.88 years (SD = 6.2) were included in the study. The most important characteristics of the participants can be consulted in Table 1.

**Table 1:** Socio-demographic characteristics of participants.

**Participants**	**Source of data**	**Age**	**Gender**	**Marital status**	**Autonomous community of qualification**	**Year of completion of specialty**	**Time working in primary care (including residency period)**
1	Focus group	32	Female	Single	Madrid	2020	3 years and 9 months
2	Focus group	35	Female	Single	Madrid	2020	3 years and 9 months
3	Focus group	51	Female	Single	Madrid	2020	3 years and 9 months
4	Focus group	27	Female	Single	Madrid	2020	3 years and 9 months
5	Interview	27	Female	Single	Madrid	2021	2 years and 9 months
6	Interview	25	Female	Single	Madrid	2021	3 years
7	Interview	26	Female	Single	Castilla-La Mancha	2020	3 years and 8 months
8	Interview	28	Female	Single	Madrid	2019	6 years
9	Interview	26	Female	Single	Castilla-La Mancha	2021	4 years
10	Interview	31	Female	Single	Madrid	2018	5 years
11	Interview	27	Female	Single	Madrid	2020	2 years and 7 months
12	Interview	28	Female	Single	Madrid	2018	3 years
13	Interview	31	Female	Single	Madrid	2019	4 years
14	Interview	27	Female	Single	Cataluña	2021	4 years
15	Interview	28	Female	Single	Madrid	2018	5 years and 6 months
16	Interview	29	Female	Single	Madrid	2019	5 years and 6 months

### 2.3. Data collection

Data collection was made through 12 individual semi-structured interviews and a focus group of 4 nurses. Data saturation was reached with 12 participants and confirmed with the remaining participants (20). The questions were developed based on the literature review on specialty training. The interviews and the focus-group discussion were conducted by two study investigators using the Zoom platform due to the COVID 19 pandemic and the geographic location of interviewees. One of the researchers conducted the interviews, and the other took notes in a field notebook. The individual interviews lasted an average of 45 mins. The duration of the focus group was 60 mins. The development of both was recorded with the consent of the participants and later transcribed by the researchers. All the participants accepted to answer questions once the interviews had been transcribed. Table 2 shows the protocol and script of the interviews.

**Table 2:** Interview protocol.

**Phase**	**Development**	**Content/Sample question**
Beginning of the interview	Reasons	As Family and Community Nurse Practitioners, we would like to hear about your experiences of the training you have received in your specialty.
	Ethical issues	Participation is completely voluntary, and withdrawal from the study is at your discretion. The sessions will be recorded and subsequently transcribed. Your identity will be protected, and your name and personal data will not be disclosed.
	Introductory question	Describe your experience as a nurse in Family and Community Nursing in recent years?
Development of the interview	Conversation guide	Describe your specialty training during your residency years? What do you think about it? Do you think that more training is needed during the residency period for Family and Community Nurse Practitioners? What is your opinion? Do you think you have acquired the necessary competencies, and why? How prepared do you think you were when you finished your residency? What training would you add or eliminate? Why?
	Final question	Would you like to say anything else that you think is interesting and we have not referred to?
Closing the interview	Acknowledgments	Thank you very much for your participation and for dedicating your time to this research. Your input is very important for the study and will be very helpful.
	Proposal	We would like to remind you that you can contact us if you feel you have anything else of interest to add at a later date. After finishing the transcription process of the interviews we will show them to you, and once the study is finished we will show you the results.

### 2.4. Data analysis

The transcripts and field notes were entered into the computer software. Data analysis was performed by two researchers, following a thematic analysis method that contained a series of phases (21): (1) Familiarization with the data: the transcripts were read by the researchers to understand everything the participants said. (2) Systematic data coding: the most significant quotes were selected and assigned codes using the “*in-vivo* coding,” “open coding,” and “apply codes” functions in ATLAS. ti. 9. (3) Generation of initial themes from the coded data: initial themes were generated by grouping codes that shared patterns of meaning and had a meaningful relationship around a central idea. (4) Theme development and review: all generated themes and the quotations on which they were developed were double-checked for consistency with the codes they included. (5) Detail, designate, and delimit themes: the researchers reviewed the final themes, refined them, and created the final names for the themes. (6) Report writing in preparing this research report: the most demonstrative citations were selected. Finally, the researchers clarified the report by filtering out the essential fragments and relating them to the literature review and the aims of the study.

### 2.5. Rigor

The quality criteria of Lincoln and Guba (22) were followed in this descriptive qualitative study. Credibility: the process by which the data were collected was described in detail. In addition, the analytical process was carefully checked by two independent reviewers. Transferability: the method, participants, setting, and context of the study were thoroughly described. Dependability: the study methodology was described in detail. Reliability: two investigators who were not involved in data collection verified the data analysis. Confirmability: the analysis of the interviews was sent by email to the participants to validate the results and confirm their responses.

## 3. Results

Two main themes and six subthemes were obtained from the data analysis, and these allowed us to describe the experiences of FCNPs during specialty training (Table 3).

**Table 3:** Themes, subthemes, and units of meaning.

Theme 1. The residency period, more than a training.	1.1. Training during the FCNP residency period.	Complete training, different training, extra training, tough process, lack of quality training, lack of quality trainers.
	1.2. Specialization through constant struggle.	On-call, mentoring, versatile girls, lack of knowledge of the specialty, struggle with the medical teaching unit, hospital, and on-call tutors, family at the health center.
	1.3. Moderate optimism about the future of the specialty.	Pessimistic future, improvements in the future, changes in nursing management, exceptional pathway, specific exchange, specific competitive examinations.
Theme 2. A journey from illusion to disappointment.	2.1. Beginning of the residency: feeling special.	Feeling happy, emotions, motivation, feeling overwhelmed, feeling special.
	2.2. During the residency: between satisfaction and incomprehension.	Feeling happy, comfort, anxiety, satisfaction, overloaded, feeling located, dependence, feeling lost.
	2.3. At the end of the residency: power and frustration.	Lack of comprehension, feeling powerful, frustration, hope, commitment, undervalued, loneliness, feeling prepared, disappointment, feeling independent, worth.

### 3.1. The residency period, more than a training

This topic described the training that the FCNPs received during the training period. They considered the residency an excellent training opportunity although they described it as a tough process in which they had to constantly struggle to be trained as specialists. Despite this, they reported not feeling recognized as specialists, but they were optimistic and expected positive changes for the specialty in the future.

#### 3.1.1. Training during the FCNP residency period

The participants perceived that the training during the residency period was useful, positive, and wellplanned, and could not be achieved with any other type of training. However, several of the participants reported that the research-based training was deficient and considered that more importance should be given to evidence-based nursing and research training for nurses in the training plan for the FCNP specialty.

“*With a degree you can't even get the same training, not even with a Master's degree, not by a long shot”* (P5).

“*I consider that in general I have acquired the necessary competencies with the exception of training in research”* (P11).

They also suggested that the interdisciplinary courses could be updated or modified. They considered it interesting to add more practical courses related to Family and Community Nursing skills. They detailed courses such as management of chronic diseases, basic notions of nutrition, electrocardiograms, wound management, bandages, or more specific situations in consultation or at home, and even communication skills with adolescents with mental illnesses. Likewise, they gave great importance to updating training in education, prevention and health promotion, and insisted that the community nursing part was somewhat deficient and that they themselves should seek complementary training.

“*I would also give more weight to the community part, which I think is given little weight. This seems to be the catchphrase, but it is the complete specialty”* (P15).

They also insisted on the lack of quality in certain courses or rotations, giving three reasons for this: the perceived lack of motivation and training of some trainers; the organization of the teaching units, which are much more medical than nursing, and the differences in training between teaching units and autonomous communities.

“*In quantity of training I would not add anything, but rather in quality…”* (P9).

The FCNPs referred that a fundamental pillar on which their training depended were their tutors at the health center and the trainers they met in the different rotations and courses. It was they who transmitted their knowledge and new updates to them daily during the training. And they specified that if the trainer was well qualified and motivated, they transmitted exceptional knowledge to them. On the contrary, if the trainer was unmotivated or was not an expert in the subject, the opposite was true. As a result, they perceived that their training greatly depended on the preparation and motivation of their trainers.

“*My experience in Madrid was very good because my center was accredited with specialist tutors”* (P10).

“*Many times the training depended on what the tutor did, and we know that there is also a lot of variability”* (P8).

#### 3.1.2. Specializing through constant struggle

Similarly, they described the specialty period as a tough process. They attributed this to the low salary they received and the large workload during the residency period: care work, training, emergency room shifts, and different rotations.

“*The specialty seems tough to me because we work long hours… and all for a very low salary”* (P13).

Most of them focused on the difficulty of the emergency room shifts and the lack of supervision that exists in this rotation. They said that they should be supervised, but, during this rotation, most of the participants found supervision non-existent. In addition, they perceived that training in the emergency department was limited to mere nursing techniques and did not feel that it added value to their specialty. However, they insisted that the assignment of a formal tutor specializing in emergency medicine would allow them to learn not only the techniques but also to consolidate concepts. And they described that when this was not the case, the emergency rotation was poorly focused training in which they simply felt they were cheap labor.

“*What I wanted to emphasize is that the shifts basically do not provide any knowledge because they are poorly focused… we go to an emergency department where it is very difficult to find a specialist tutor in emergency medicine”* (P1).

They also detailed the lack of knowledge of their role especially in hospital rotations, where they generally found themselves more neglected. One of the things that surprised them the most was the lack of knowledge of the competencies of the FCNPs among their colleagues, many of them nurses, resident interns, and attending physicians. They pointed out that on some occasions even the rotation tutors themselves did not know what the role of the FCNP residents was. All this lack of knowledge made learning difficult.

“*It is totally unknown to people. And not only by the people in the street but also professionals…”* (P10).

“*… and I was surprised when they asked me what the EIR* [Resident Intern Nurse] *was”* (P12).

On the other hand, they said that this does not usually happen in their reference Health Centers, where coworkers had much greater knowledge of the role of the FCNP resident, indicating that this helped their training. In addition, they said that the tutors for this rotation were usually more involved and encouraged their training in fields such as health education, community interventions or research, helping and giving them information about participation in courses and congresses.

“*My tutor at the Health Center was a woman standard bearer for nursing and was very supportive, and she was very attentive to my residency”* (P14).

#### 3.1.3. Moderate optimism about the future of the specialty

Participants were moderately optimistic about the recognition of their specialty. Hopeful participants mentioned evolutionary nursing and believed that specialties would be the basis for the future of nursing. But they equally perceived that at present this was complicated.

“*I hope (that the EFyC* [Family and Community Nurse] *will be recognized) with all my heart”* (P13).

“*I think we are going to get better, but it's a very slow process”* (P6).

They mentioned that successive generations of new residents have already perceived positive changes in the training provided by the Teaching Units. And they compared the current situation of nursing specialties to what previously happened with physicians.

“*There are some (rotations) that they have removed because they did not contribute anything to us and others that they have added”* (P5).

“*I believe that our situation will improve as it did with the physicians”* (P9).

But for this to happen and to be able to achieve the advancement of the specialty, the FCNPs said that it is the nursing colleagues themselves who must make the initial advances, raising awareness and emphasizing the role of the specialist nurse to the nursing area, in addition to making changes in the management and leadership of nursing, salary recognition, and the creation of exchanges and specific competitive examinations for specialist nurses.

“*Recognizing specialties, putting into effect the specific exchanges, encouraging people to apply for the EIR, clarifying competencies, supporting training, and improving it every day by adapting it to the new health trends”* (P7).

And they believed that, if this happens, by increasing the number of FCNPs they will cease to be a minority, which would also favor the development of the specialty.

“*There are more of us and we are really starting to be recognized”* (P16).

### 3.2. A journey from excitement to disappointment

This theme described the feelings expressed by the nurses specializing in FCNP from the beginning of their residency to its completion in order to enter the world of work. The participants related the desire to specialize with commitment to their vocation. They also insisted that although they considered nursing to be a vocational profession, they were not heroines but rather people with their own lives and personal relationships that should be respected.

#### 3.2.1. Beginning of the residency: feeling special

At the beginning of the residency, most of the participants felt overwhelmed by a new experience which for many of them it was not only a change of work but also a personal one. Participants reported that they had to move from home and loved ones in order to specialize. Nevertheless, they felt excited and very motivated. In addition, they were very proud of having passed such a complicated test as the EIR exam and were thus able to opt for a place to train as a specialist, which was not achieved by all applicants in the test.

“*It was a lot of things together…So I had a first month of adaptation that was somewhat overwhelming”* (P15).

They found themselves motivated and referred to their desire to learn, to do research, to absorb all that they would discover during the training, to grow as an FCNP. They even thought that their opinion could be important to modify certain things and improve the training, such as adding or eliminating certain courses and rotations through different services related to the specialty and external rotations.

“*I felt motivated and happy as I had achieved my goal”* (P8).

#### 3.2.2. During residency: between satisfaction and incomprehension

At this stage the participants described sensations ranging from happiness and satisfaction to incomprehension, passing through a mixture of feelings between comfort and being overwhelmed. They perceived that in many situations they felt overwhelmed and overburdened, and even experienced anxiety at certain points. The excessive hours of the workday, courses, and the end-of-residency research project were situations that pushed some of the participants to the limit.

“*The last few days I wanted to quit. I was super saturated”* (P6).

On the other hand, most of them agreed that certain rotations, especially in hospital rotations whose emergency departments were at the top of the ranking, they felt out of place and lost. Even the participants in this study themselves did not know what their role really was at the beginning of their training.

“*When I started residency at the beginning, I didn't really know what my competencies were as a resident”* (P5).

“*It is not that I was uncomfortable in the hospital, but they frequently failed to understand what our role was”* (P16).

On the contrary, they expressed the comfort they felt when they were in the health centers where their tutors were located. They normally considered the coworkers, the rest of the residents, and the tutor to be as a real family.

“*At the health center you are more like family”* (P16).

They also explained that on many occasions they had felt dependent on the tutors and/or the Teaching Unit. They came to feel undervalued. They felt that how they should act or work was imposed without considering their opinion even if they did not agree. They felt they had to justify all their actions in order to be taken into account in decision-making, which was not enough on several occasions. But in spite of this, they gradually felt more comfortable in their situation as the residency period went on, and in general they felt happy and satisfied with their learning progress both professionally and personally.

“*You have to insist all the time… and in any discussion, as a resident, I've always had to challenge what's been said”* (P5).

“*I was satisfied with the decision I made to start it [the training]”* (P13).

#### 3.2.3. At the end of the residency: power and frustration

This subtheme describes the feelings of the participants once the training period was over and they had obtained the title of specialist in FCNP. They agreed that they felt empowered and independent, with the full capacity to be able to solve their patients' problems and with the security of knowing most of their competencies in primary care. They were proud of themselves for having achieved their goal and felt prepared to work in their job as specialists but also felt hurt as they did not feel that the administration recognized them as specialists or helped them to develop all their skills.

“*I think we have a very powerful relationship with people and a lot of power to make changes in society”* (P8).

“*It is very sad and painful that a profession as important and valuable as ours is not recognized”* (P13).

Before starting their specialty, they felt committed to the population, and after the course in FCNP this feeling remained. However, they reported feeling somewhat limited in their work as specialists. Because of this, they often described feeling frustrated, wasted, and stagnant because, although most of them managed to work in Primary Care, they were not able to develop all their competencies as specialist nurses but rather covered back-up positions and were not able to exploit their full potential.

“*I have managed to work in Primary Care, which was what I wanted, but there are certain barriers that prevent us from doing our job 100%”* (P16).

Despite all these mixed feelings, most of the participants were hopeful that in the future their specialty will advance, they will be able to develop all their specialist competencies and they will be truly recognized. However, they felt that it was worthwhile to specialize not only for the increase in knowledge, but also for their personal growth, and they felt very good about what they had done.

“*I would definitely do it again… it has allowed me to acquire a lot of knowledge and have a fluency behind the desk that I would not have obtained otherwise, and well… also for personal growth”* (P4).

## 4. Discussion

The aim of this study was to describe and understand the experiences of nurses during their training process in the specialty of FCN in Spain. This study is not a nationwide representative study, and it is restricted only FCNPs who have been trained in the communities of Castilla. The participants described that this training period was harder than usual and they had to constantly overcome obstacles, and, on the other hand, it was like a journey where they went from illusion to disappointment with the profession. The training was a unique learning opportunity in which they became nurses qualified to perform their work (12). This fact can be observed in several studies, where primary care nurses are trained in multiple competencies and feel prepared to perform their duties (6, 10). Thus, the study by Guo et al. described how community nurses who had not received specific training felt less prepared and more stressed in their care work (23).

They also thought that, although the training plan of the FCNP residency provided them with excellent training in FCNP skills, they considered that a series of changes in rotations and courses that would provide them with more knowledge in topics related to the specialty should be made. One of the problems commented by the participants was the absence of content related to research. However, in contrast, Hernández-Crespo et al. pointed out the low number of hours of training in Public and Community Health topics taught in the teaching units compared to clinical and research topics (24). This could be due to the fact that each training unit teaches different contents, so it would be necessary to unify the concepts to be taught. Miranda Neto considers that training plans during residency should be well defined and are an opportunity to train specialist nurses in the competencies needed to provide advanced care in primary care (25). In this sense, it is necessary to train specialist nurses with new teaching methodologies such as clinical simulation and provide them with greater empowerment and self-efficacy in the work they must perform (26). During FCNP training, the methodologies applied were classroom teaching and clinical practice.

On the other hand, the FCNPs felt motivated and satisfied with the training although they were also overloaded by the heavy workload they had to put up with in primary care, which caused them some discomfort (27). This overload of patients could be solved by shiring new FCNP. The participants were committed to their work but insisted that their lives were frequently not respected and the administration took advantage of their participation without even acknowledging it (28). These facts cannot be compared with other studies as no research has been found describing the feelings or sensations experienced by the specialist nurses during their training in primary care.

These nurses are moderately optimistic about their future. They feel that the specialty is not recognized neither by the population in general nor by their peers (16, 17). They relate this situation to the lack of knowledge that the specialty suffers due to the lack of clarity of its role (3, 6). Various authors have highlighted the absence of a consensus on the definition and the use of different standardized terms around the designation of FCNPs at the international level (3, 7). Further progress on this topic could strengthen the development of standardized definitions and make their role visible to society and healthcare administrations (29). A best way to promote FCNP socially is by solving patients' problems. However it is also necessary to demonstrate the FCNP's functions and this social usefulness to the politicians who fund and regulate the services, and to the managers who run them. Lack of appreciation and feeling undervalued led our participants to demotivation, which could have an impact on the quality of care provided (30). Benefits in quality of care and efficiency of community nurse specialists compared to other groups have also been shown. The implementation of quality nursing care leads to efficient nursing care with a reduction in the number of hospital stays and in the consumption of resources (31, 32).

The participants in this study felt that in the not-so-distant future the nursing profession and its specialties will advance as has happened with the medical profession (9). However, for this to happen, the nursing colleagues themselves must also evolve and advance in the recognition of the specialty (2). Vanhook et al. consider that the key to the favorable evolution of the FCNP lies in changes in undergraduate training, specialized training, and in administration (33). For this, it is necessary that jobs are defined, and specific work grants are created for recruitment (2).

### 4.1. Limitations

This study has several limitations: Firstly, it was carried out with FCNPs who have been trained only in the communities of Castilla—La Mancha, Girona, and Madrid due to the offering of the FCNP specialty in these communities. Even so, it was important for us to know the situation of specialist nurses in the Spanish context. Secondly, the participants are all women, and this may have influenced the results. However, rather than being a limitation, it is also a reality since the nursing profession is primarily a female profession. Finally, the different data collection techniques were carried out in online format, and therefore non-verbal language could not be clearly seen. On the other hand, scientific rigor has given credibility to the study.

### 4.2. Future lines of research

Future research could describe the experiences of FCNPs once they have completed their training when they enter the world of work. In addition, it would be necessary to know the perceptions of other health professionals with respect to this group, and/or the perceptions of the patients themselves, in order to seek recognition of this training by coworkers, patients, and even political leaders.

## 5. Conclusions

The participants stated that specialization in FCNP was a unique training opportunity in which they became highly skilled specialist nurses in the performance of care. During the training they faced several obstacles. They were happy and proud to become FCNP nurse specialists although after completing the training they were frustrated due to the lack of recognition of their work, related to the lack of knowledge of nursing specialties. The results of this study demonstrate that it is necessary to continue working on unifying and improving the training received by FCNPs at the national and international levels. It is important to include in the training programs for residents curricular content that is adapted to the specialty. Education systems should therefore strengthen training to ensure that FCNP specialists acquire the necessary competencies to perform the healthcare work for which they have been trained. In addition, it is a priority to give greater visibility to FCNP by health organizations and for it to acquire greater recognition.

## Data availability statement

The original contributions presented in the study are included in the article/Supplementary material, further inquiries can be directed to the corresponding author.

## Ethics statement

The studies involving human participants were reviewed and approved by Department of Nursing, Physiotherapy and Medicine, University of Almería. The patients/participants provided their written informed consent to participate in this study.

## Author contributions

FS-M and MR-F: conceptualization and study design. FS-M, MR-F, IF-M, and MV-M: data collection, data analysis, and manuscript writing. JG-M, IS-G, and SN-N: interpretation and study supervision. All authors contributed to the article and approved the submitted version.
